# Congenital cytomegalovirus infection in newborns suspected of congenital rubella syndrome in Iran: a cross-sectional study

**DOI:** 10.1186/s12887-023-04502-3

**Published:** 2024-01-10

**Authors:** Negar Mirsalehi, Jila Yavarian, Nastaran Ghavami, Maryam Naseri, Farshad Khodakhah, Somayeh Shatizadeh Malekshahi, Sevrin Zadheidar, Talat Mokhtari-Azad, Nazanin-Zahra Shafiei-Jandaghi

**Affiliations:** 1 Virology Department, School of Public Health, Tehran University of Medical Sciences, Tehran, Iran; 2 Department of Virology, Faculty of Medical Sciences, Tarbiat Modares University, Tehran, Iran

**Keywords:** Human cytomegalovirus, Congenital rubella syndrome, Avidity assay, Genotype

## Abstract

**Background:**

Following rubella virus control, the most important cause of congenital infections is human cytomegalovirus (HCMV). Congenital CMV (cCMV) may happen both in primary and non-primary maternal infections. The present study aimed to screen cCMV in symptomatic newborns suspected of congenital rubella syndrome (CRS) in Iran.

**Methods:**

Out of 1629 collected infants' serum samples suspected of CRS but negative for rubella IgM, 524 samples were selected regarding cCMV complications. These samples were divided into two age groups: 1- one month and younger, 2- older than 1 month up to one year. Anti-HCMV IgM detection was performed on these serums. Then HCMV IgG avidity assay and HCMV DNA detection were carried out on all samples with positive and borderline results in IgM detection.

**Results:**

Herein, 3.67% of symptomatic infants aged one month and younger had positive and borderline HCMV IgM, 12.5% of which had a low avidity index (AI). HCMV IgM detection rate among symptomatic infants older than one month to one year was 14.5%. Identified genotypes in this study were gB-1(63.63%), gB2 (18.18%), and gB3 (18.18%), respectively.

**Conclusions:**

This comprehensive study was performed on serum samples of symptomatic infants clinically suspected of cCMV from all over Iran. There was a good correlation between serology findings and PCR.

## Introduction

Human cytomegalovirus (HCMV) is a host-restricted member in the β herpesvirinae subfamily of the *Herpesviridae* family [1]. Like other herpes viruses after primary infection, the virus remains latent. The rate of seroprevalence in adults is around 45% to 100% worldwide. HCMV can be transmitted both vertically and horizontally [2]. Transmission from the mother to the fetus or newborn may occur during pregnancy, at birth time, and postnatal. Horizontal transmission happens through close contact with contaminated saliva, urine, feces, blood, sexual contact, and organ transplantation [3]. Postnatal HCMV infection is acquired via interaction with cervical secretions during birth, breast milk, blood transfusion, or bodily fluids of infected persons. Approximately 9–88% of seropositive women shed HCMV into their milk, and roughly 50–60% of the newborns who have been fed contaminated breast milk became infected [4]. During pregnancy, HCMV is transmitted from the mother to the fetus in approximately 35% of gestations with maternal primary infection [5]. Intrauterine transmission of HCMV may also happen in women with prior antibodies to HCMV either by reactivation of previous maternal infection (recurrent infection) or by the acquisition of a different viral strain (re-infection). For these non-primary infections the proportion of vertical transmissions is roughly 1.1 to 1.7% [6].

In newborns, postnatal HCMV infection is usually asymptomatic [7]; however, prenatal infection may cause devastating abnormalities [2]. In developed countries following the control of rubella virus circulation, the most important cause of congenital infections is HCMV [1], with an estimated incidence rate of 0.5–2% in all live births [8]. In developing countries, the rate of congenital infection is around 2–4% [9] and 6–14% [10] in different studies. The virus can replicate in the placenta, contaminate the fetus, and cause congenital CMV (cCMV) and abnormalities in the fetus [11]. So, cCMV may happen both in primary and non-primary maternal infections but with different incidence rates [12]. Most of the cCMV infections (CCI) [12] (75–90%) are asymptomatic at birth. More or less than half of the symptomatic infants are small for their gestational age, and one-third are born prematurely. The most common observed clinical findings are petechial rash, jaundice, hepatosplenomegaly and neurologic abnormalities [13]. Mental retardation, seizures, speech delay, learning disabilities, chorioretinitis, optic nerve atrophy, and defects in dentition are the other most common long-term complications in infants with cCMV [14, 15]. To diagnose cCMV in suspected newborns up to roughly 3 weeks after birth, the standard technique is HCMV isolation from the body fluids (such as urine, blood, saliva and cerebrospinal fluid) using cell culture. However, virus isolation is not generally used for cCMV diagnosis, as it is time consuming and expensive. The recommended and common methods are the detection of HCMV-DNA and anti-HCMV specific IgM [16, 17].

Nucleotide variability was determined for about 20 open reading frames (ORFs) of HCMV encoding, glycoproteins B (gB), gH, and gN, as well as tumor necrosis factor (TNF)-α receptor (UL144). In glycoprotein B which is the major HCMV envelope protein composed of 906 amino acids, the regions between 448 and 481 codons were defined as the highly polymorphic site. Four main HCMV genotypes, gB1, gB2, gB3, and gB4, and three rare non-prototypic variants, gB5, gB6, and gB7, were defined based on this area [18]. HCMV molecular genotyping can provide insights into HCMV diversity within an individual host. Different strains of HCMV may have varying levels of virulence, and genotyping can help identify which strains are more likely to cause severe disease [19]. There is no clear consensus on whether there is an association between HCMV genotype and specific clinical presentation. Some studies have found no significant association between specific genotypes and clinical features [20], while others have found associations between certain genotypes and specific symptoms of cCMV infections [21, 22].

The present study aimed to screen cCMV using HCMV specific IgM in newborns suspected of CRS in Iran. Our secondary goal was to differentiate the primary and non-primary maternal infections using IgG avidity assay in newborns who were HCMV IgM positive. Furthermore, probable congenital infections, perinatal and postnatal infections were also investigated for infants older than 1 month of age, using the mentioned methods. Finally, the genotypes of detected HCMV strains were investigated.

## Methods

### Study design, patients and samples

For congenital rubella surveillance, the Iran Ministry of Health collects clinical samples of all suspected infants younger than one-year-old from all over the country. These samples are sent to Measles and Rubella National Laboratory in Virology Department, School of Public Health, Tehran University of Medical Sciences**.** During 2016 and 2017, altogether 1629 serum samples of infants suspected of CRS aged 3 days up to one year were collected. It should be noted that all of these samples were negative for rubella specific IgM. Considering the similarity and some difference between the symptoms of CRS and cCMV, among these specimens, 524 serums of symptomatic infants, who could be suspected of cCMV based on the symptoms and complications, were selected and assessed for HCMV infection. Based on the infants’ ages, their serum samples were divided into two groups. In the first group, there were 244 serum samples of symptomatic infants aged 1 month and younger. In the second group, there were 280 serum samples from infants aged older than 1 month up to one year.

### Serology

Anti-HCMV IgM detection was performed on 524 serum samples using a commercial enzyme-linked immunosorbent assay (Anti-CMV ELISA-IgM, EuroimmunLubeck, Germany). The interpretation of IgM results, according to the kit’s instruction was as follows: Ratio < 0.8 was considered negative, ratio ≥ 0.8 to 1.1 was considered borderline and ratio ≥ 1.1 was considered positive.

Then for both groups of infants HCMV IgG avidity assay was carried out on all IgM positive and borderline cases using a commercial kit (Anti-CMV ELISA-IgG Avidity, Euroimmun Lubeck, Germany). Avidity results were calculated and interpreted according to the kit manual as follows: Avidity index (AI) > 40% was considered as low avidity IgG indicating primary CMV infection. AI 40–60% was considered as moderate avidity (equivocal) and AI > 60% was considered as high avidity antibody indicating past CMV infection [23].

### HCMV molecular detection and genotyping

HCMV DNA detection was performed on all samples with positive and borderline results in IgM detection**.** At first, DNA was extracted using the High Pure Viral Nucleic Acid Kit (Roche, Germany) according to the manufacturer’s instructions. The extracted DNA was stored at -20˚C before being used as a template to detect CMV DNA. Then, a semi-nested PCR reaction was applied to detect CMV DNA by using specific primers for a part of the gB gene (UL55 region) from a previous study [24]. DNA amplification was performed in 50 μl total reaction volume in the first and 50 μl in the second round. The first reaction contained: 5 μl PCR Buffer, 2 μl Mgcl2, 1.5 μl dNTP, 1.5 μl forward and 1.5 μl reverse primers, 28 μl deionized water, 0.5 μl Taq DNA polymerase and 10 μl target DNA. In the second reaction the volume of deionized water was 33 μl and the target DNA was 5 μl (Table 1). For virus type identification in HCMV positive cases, the PCR products of the second round were purified and subjected to Sanger sequencing in forward and reverse directions. Sequencing reactions were performed using the ABI Big Dye Terminator Cycle Sequencing Kit and a 3130 Genetic Analyzer (Applied Biosystems). For genotype identifications, a phylogenetic tree with 1000 bootstrap was constructed using MEGA 10 software based on the maximum likelihood method via the Tamura-Nei model.

**Table 1:** PCR mix and condition for HCMV molecular detection and genotyping by Semi-nested PCR

**First round PCR mixture**		**Second round PCR mixture**		**Amplification**	Temperature	Time
Component	Volume	Component	Volume	Pre-Denaturation	95◦C	5'
DDW	28 μl	DDW	33 μl	Denaturation	94◦C	1'
Buffer	5 μl	Buffer	5 μl	Annealing	55◦C	1'
Mgcl2	2 μl	Mgcl2	2 μl	Extension	72◦C	1'
dNTP	1.5 μl	dNTP	1.5 μl	Final extention	72◦C	8'
Forward Primer	1.5 μl	Forward Primer	1.5 μl	Hold	4◦C	-
Reverse 1 Primer	1.5 μl	Reverse 2 primer	1.5 μl			
Taq DNA polymerase	0.5 μl	Taq DNA polymerase	0.5 μl			
Total	50 μl	Total	50 μl			

### Statistical analysis

Continuous and categorical variables were shown as mean (SD) and n (%), respectively. To examine differences between independent groups, the χ2 test, or Fisher's exact test is applied where appropriate. A two-sided α of less than 0·05 was considered statistically significant. Statistical analyses were performed using SPSS version 22.

## Result

### HCMV serology and genome detection results in infants aged 1 month and younger

Two hundred thirty-eight of these 244 infants had gender information as follows: 124 (52.10%) were female and 114 (47.89%) were male (Table 2). IgM detection, IgG avidity evaluation and PCR were performed. The result of HCMV IgM detection test on these 244 serum samples showed that 5 (2.04%) cases were positive, 4 (1.63%) cases were borderline and 235 (96.3%) cases were negative. Totally, the rate of IgM detection which was considered as CCI [12] in these infants was estimated 3.67%. IgG avidity index (AI) was measured in positive and borderline serums for HCMV IgM (8 out of 9, one sample was excluded due to inadequate serum sample). The result showed 7/8 (87.5%) had high AI and 1 (12.5%) had low AI (Table 3).

**Table 2:** Comparison of infants suspected of cCMV aged 1 month and younger based on gender

Year	Gender		Total	Chi-Square Tests	*P* value:
	Female	Male			
2016	42 (56.0%)	33 (44.0%)	75 (31.51%)		0.46
2017	82 (50.30%)	81 (49.69%)	163 (68.48%)		
Total	124 (52.10%)	114 (47.89%)	238 (100%)

**Table 3:** IgG avidity in symptomatic infants suspected of cCMV aged 1 month and younger

Year	CMV IgM	N	IgG Avidity		
			Low AI N (%)	Equivocal AI N (%)	High AI N (%)
2016	Positive	2	----	----	2 (100%)
	Borderline	1	1 (100%)	----	----
2017	Positive	3	----	----	3 (100%)
	Borderline	2	----	----	2 (100%)
Total	Positive	5	----	----	5 (100%)
	Borderline	3	1 (33.33%)	----	2 (66.66%)
	Positive and Borderline		1 (12.5%)	----	7 (87.5%)

The evaluation of PCR results in these 8 samples showed that HCMV-DNA was detected in 2 serums.

### HCMV serology and genome detection results in infants older than one month to one year

Two hundred eighty serum specimens were collected from infants aged older than 1 month to 12 months.

Of these 280 infants, 130 were female and 150 were male (Table 4). Evaluation of IgM detection in these infants showed: 19 (6.7%) serums were positive, 22 (7.8%) were borderline and 239 (85.5%) cases were negative. One IgM-positive sample was not evaluated for avidity due to the insufficient amount of serum. Out of 40 positive and borderline IgM serums among mentioned infants 8 (20%) had low AI, 16 (40%) had equivocal AI and 16 (40%) had high AI (Table 5). For all positive and borderline IgM serums, PCR testing was done, in which 9 cases were positive.

**Table 4:** Comparison of infants suspected of cCMV older than 1 to 12 months of age based on gender

Year	Gender		Total
	Female	Male	
2016	55 (42.3%)	75 (57.7%)	130 (46.42%)
2017	75 (50%)	75 (50%)	150 (53.57%)
Total	130 (46.42%)	150 (53.57%)	280 (100%)

**Table 5:** IgG avidity in symptomatic infants suspected of cCMV older than 1 to 12 months of age

Year	CMV IgM	N	IgG avidity		
			Low AI N (%)	Equivocal AI N (%)	High AI N(%)
2016	Positive	7	2 (28.5%)	2 (28.5%)	3 (42.8%)
	Borderline	8	2 (25%)	5 (62.5%)	1 (12.5%)
2017	Positive	11	2 (18.1%)	3 (27.7%)	6 (54.5%)
	Borderline	14	2 (14.2%)	6 (42.8%)	6 (42.8%)
Total	Positive	18	4 (22.2%)	5 (27.7%)	9 (50%)
	Borderline	22	4 (18.1%)	11 (50%)	7 (31.8%)
	Positive and Borderline		8 (20%)	16 (40%)	16 (40%)

### Genotypes of HCMV

**Fig. 1:**
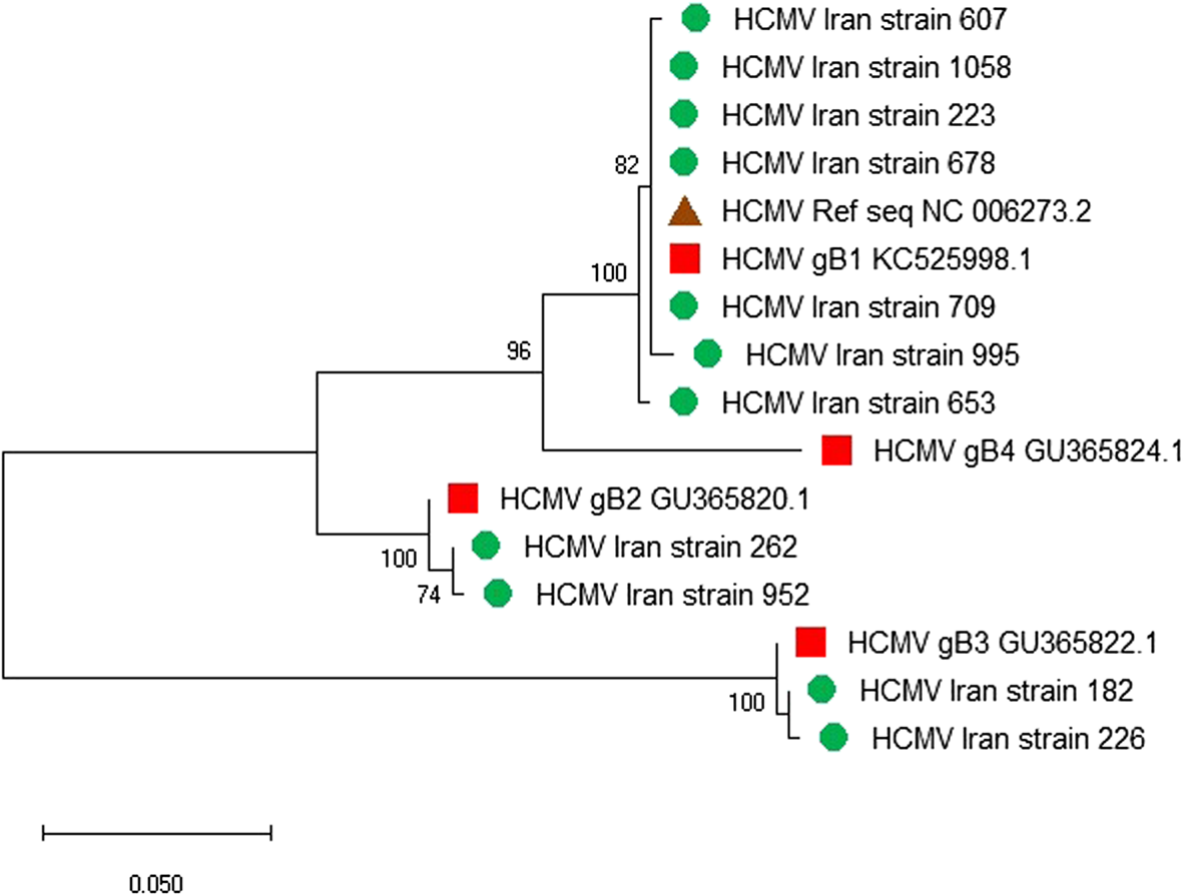
HCMV gB gene of strains from Iran compared with the HCMV reference sequences displayed in a phylogenetic tree determined using the maximum likelihood method via Tamura-Nei model with MEGA 10 software. Only bootstrap values greater than 70% are displayed at the branch nodes. The genotypes of samples from Iran are indicated as solid circle. Sequences of Iran fell within gB1, gB2 and gB3 genotypes

Overall, 11 cases of HCMV detected genomes in this study could be successfully genotyped. Herein, among four major gB genotypes, gB-1, gB-2 and gB-3 were detected (Fig. 1). The most frequent genotype was gB-1(63.63%). Then, gB2 (18.18%) and gB3 (18.18%) were found. Of the 2 identified strains in one month old and younger infants, one belonged to the gB1 genotype, which was collected from Khuzestan province and the other one belonged to gB3 genotype collected from Tehran province. In infants older than one month to one year, out of 11 strains, 9 strains were sequenced properly. The results showed, 6 cases (66.67%) belonged to gB1 genotype from Tehran, Isfahan, Alborz, and Mazandaran provinces, 2 (22.2%) was gB2 genotype from Azerbaijan Sharghi and Khorasan and one (11.1%) was gB3 genotype from Azerbaijan Sharghi province.

## Discussion

The half-life of IgG antibodies is approximately 21 to 26 days and maternal IgG generally disappears by 4 months of life [25]. It should be noted that, low AI among infants aged 1 month and younger indicates a certain maternal HCMV primary infection. High AI suggests a non-primary maternal infection but it should be considered that some mothers may have had primary infection in the first months of pregnancy. For older children, low AI indicates a current HCMV infection, while high AI reveals a past HCMV infection.

This study showed that in Iran during 2016–2017, the incidence rate of CCI in symptomatic infants aged 1 month and younger was 3.67%. It is important to note that congenital rubella was negative in these infants.

In several studies conducted in Iran and other countries, the incidence rate of CCI in neonates and infants was evaluated using different methods A cross-sectional study in Birjand, Iran in 2018 showed that the rate of CCI in randomly selected neonates (868 cases) using PCR on saliva was 1.61% [26]. A study in Tehran, Iran in 2017 tested 100 urine samples of symptomatic infants, under 3 weeks of birth to diagnose CCI using PCR and ELISA. HCMV-DNA was detected in the urine of 58 infants and 20 serums were positive for HCMV-IgM. The prevalence of cCMV was reported 58% [27].

In a prospective study in 2016 CCI was identified in 8 (0.49%) out of 1617 urine specimens of symptomatic Iranian neonates less than two weeks of age [28].

These findings highlight the importance of early diagnosis and management of CCI in neonates and infants. Further research is needed to better understand the epidemiology and clinical manifestations of HCMV infection in different populations.

The prevalence of CCI varies considerably in developing country, ranging from 6 to 14% [10], while in industrialized countries such as Western Europe, the United States, Canada, and Australia it affects around 0.5–0.7% of all live births [29]. HCMV IgG avidity can be used to distinguish primary from non-primary infection [23]. Like our study where maternal specimens are not available, IgG avidity assay can be performed on infants' serum samples to determine whether their mothers had primary infection during pregnancy. In infants 1 month of age and younger, only 12.5% of CCI cases could be attributed to the maternal primary HCMV infection, according to the low AI results. On the other hand, 87.5% of neonates with CCI had high AI. This probably suggests that their mothers had non-primary infections. However, there is a possibility that some mothers had a primary infection in the early months of pregnancy and the avidity had matured by the end of pregnancy.

The nature of non-primary maternal HCMV infections could be re-infection with a different viral strain of HCMV, or recurrent infection from reactivation of a latent virus [30].

Studies have shown that the risk for long-term complications was higher in infants born to mothers with primary infection in the first half of pregnancy rather than non-primary infections [1]. In developing countries approximately 90% of women in childbearing age are immune to HCMV therefore HCMV reactivations occur more than primary infections [31].

In Iran, a cohort study found that 93% of child bearing aged women were seropositive for HCMV [32], while a prospective study in Iran showed that 84% of women were HCMV seropositive and the rate of seropositivity was higher in people with lower socioeconomic conditions [33]. In another study, the seroprevalence of HCMV among pregnant women in the east of Iran was 72.1% [34]. In a systematic review conducted from 2008–2017 in Iran, the pooled prevalence rate of HCMV IgG among women of reproductive age was estimated at 90%. The highest prevalence rate of HCMV IgG was found in Tehran, Rasht, Mashhad, and Yasuj, while the lowest prevalence was detected in Jahrom [35].

HCMV seropositivity in women of reproductive age ranged from 45 to > 90%, globally. HCMV seroprevalence tends to be higher in developing countries (> 90% in Brazil, 70–80% in Ghana, > 90% in India, 80–90% in South Africa and > 90% in Turkey) and lower in developed countries (40–70% in Western Europe, 60–70% in Australia, 60–70% in Canada and 50–60% in the United States) [31].

Serum samples were used for cCMV detection by PCR method although it is not the sample of choice [36]. Besides, the genotypes of detected HCMV were identified through nucleotide sequencing and phylogenetic analysis.

Herein, the prevalence of HCMV infection among infants older than one month to one year, was 14.5% which could be attributed to congenital, perinatal or postnatal infections. A study by Noor bakhsh et al. evaluated the HCMV infection in infants suspected of intrauterine infection and in controls. The study found that 41.9% (31/74) of cases had HCMV IgM and 74% (54/74) had HCMV IgG while in the control group, 6.2% had HCMV IgM and 95.4% had HCMV IgG [37].

In a long-term study between 2003 and 2015, the prevalence of HCMV IgM and IgG among 517 symptomatic newborns and children aged 1–3 months old was assessed. Among all of these cases, 97 (18.7%) were HCMV IgM positive and 438 (84.7%) were HCMV IgG positive. The rate of HCMV IgM positivity in 1–3 months old children (25.8%) was higher (fourfold) than that in newborns (6.4%) [38, 39].

As mentioned, the results of IgG avidity assay in our study showed that around 80% of symptomatic infants older than one month to one year had high or moderate avidity indicating IgG maturation in these infants due to passing time.

For all positive and borderline IgM serum samples, genome detection was performed by semi-nested PCR. In infants aged one month old and younger, HCMV genome was detected only in two cases, both of which had high avidity IgG, which were caused by non-primary maternal active infection. Of these 2 identified strains, one case (50%) belonged to gB1 genotype and the other case (50%) to gB3 genotype. Among the children of the other group, HCMV genotype identification of 9 detected strains in this study showed, six cases (66.67%) belonged to gB1 genotype, 2 (22.2%) cases to gB2 genotype and one (11.1%) case to gB3 genotype. The high frequency of gB1 genotype was consistent with other studies in Asia [40, 41].

HCMV genotyping is useful to examine potential differences in the pathogenicity of strains and to show infections with a mixture of HCMV strains involved in HCMV disease in adults and congenitally infected newborns [42]. In a study conducted in the south of Iran, HCMV genome detection was performed on 80 urine samples of NICU hospitalized neonates in two age groups (younger and older than 30 days) and only one newborn under 30 days had HCMV-DNA. So the rate of CCI was estimated 1.2% [43].

In the study conducted in Cuba on 361 newborns with clinically suspected HCMV infection it was found that 19.7% of infants had congenital infection. All of the four major HCMV genotypes were detected among the infants with the most frequent genotype being gB-2 [44].

The total frequency of cCMV infection was 18.4% among 576 suspected Indian newborns (2 weeks after Birth) with confirmed seropositive test. Between the different gB genotypes, gB1 had the highest and gB4 had the lowest frequencies [40].

This study had several limitations: The best samples for CCI diagnosis are urine or saliva that were not available. Maternal serum samples were unavailable to assess HCMV IgM and IgG.

## Conclusion

This study was a comprehensive research conducted on serum samples of symptomatic infants clinically suspected of cCMV from all over Iran. Based on maternal IgG avidity, in a few of the cases, this congenital infection was caused by a primary maternal infection while in the majority of cases could be due to a non-primary infection. In the latter group, the possibility that some mothers had primary infection in the first months of pregnancy and avidity matured at the end of pregnancy should be considered. The incidence rate of HCMV infection among infants older than one month to one year, was evaluated which could be attributed to congenital, perinatal, or postnatal infections. The genotypes of HCMV were identified, and gB-1 was the most frequent genotype.

## Data Availability

The data that support the findings of this study are available on request from the corresponding author.
